# Myocardial Infarction and Aortic Root Mycotic Aneurysm Complicating Aortic Valve Endocarditis: Utility of Cardiac CT

**DOI:** 10.1155/2016/3756302

**Published:** 2016-08-25

**Authors:** Aimee E. Moores, Michael S. Cahill, Todd C. Villines

**Affiliations:** ^1^F. Edward Hebert School of Medicine, Uniformed Services University of the Health Sciences, Bethesda, MD, USA; ^2^Cardiology Service, Department of Medicine, Walter Reed National Military Medical Center, Bethesda, MD, USA

## Abstract

Aortic mycotic aneurysms are a rare but life-threatening potential complication of infective endocarditis. Rapid deterioration of the vascular wall in highly focal areas makes these pseudoaneurysms particularly prone to rupture, resulting in uncontrolled aortic hemorrhage. While computed tomography angiography (CTA) is the imaging modality of choice for the evaluation of mycotic aneurysms, it is not routinely performed in patients with known or suspected infective endocarditis (IE). However, current valvular heart disease guidelines support the use of cardiac CTA in cases of IE and suspected perivalvular extension when there is inadequate or ambiguous visualization on echocardiography. Here, we describe a case of IE in which cardiac CTA was used for two purposes: to assess perivalvular complications and to define coronary anatomy in a patient with a suspected embolic myocardial infarction. Subsequent detection of an aortic root mycotic aneurysm not previously demonstrated on transthoracic or transesophageal echocardiography allowed for timely and uncomplicated surgical intervention, while avoiding invasive coronary angiography.

## 1. Introduction

Mycotic aneurysm (MA) is a rare, potential complication of infective endocarditis that carries significant mortality risk. The pathogenesis begins with bacterial infiltration into the vessel wall, which may occur directly through trauma, by local extension from an existing infection, or by seeding from a distant site via septic embolism or bacteremia. A robust inflammatory response ensues, resulting in rapid, focal wall degeneration [1]. Infective endocarditis remains one of the most common predisposing factors, though the incidence of MA is rare even in this case. Indeed, through 1965, only fifteen cases of MA of the aortic sinuses and ascending aorta had been reported, with sporadic case reports since then [2]. The rarity of MA has precluded the performance of randomized clinical trials and creation of evidence-based treatment guidelines; thus it presents a unique diagnostic and therapeutic challenge [1]. While transesophageal echocardiography is the standard imaging modality for infective endocarditis, it may fail to detect various perivalvular complications, including MA [3].

## 2. Case Presentation

**Figure 1:**
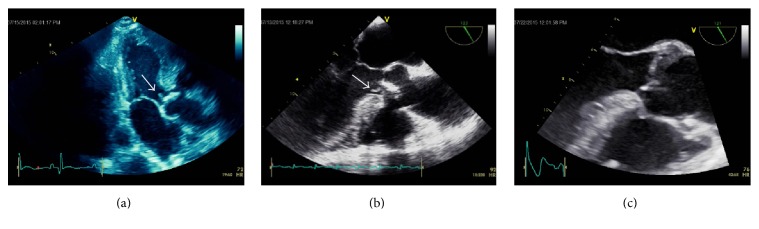
(a) Transthoracic echocardiogram image demonstrating a vegetation (arrow) on the ventricular surface of the aortic valve. (b) Transesophageal echocardiogram image demonstrating the small vegetation on the ventricular surface of the aortic valve, as well as aortic valve thickening in patient with known bicuspid aortic valve. (c) Follow-up transesophageal echocardiogram demonstrating no evidence of previously visualized vegetation, suggesting its resolution or embolism. No evidence of pseudoaneurysm was detected on serial echocardiographic studies.

**Figure 2:**
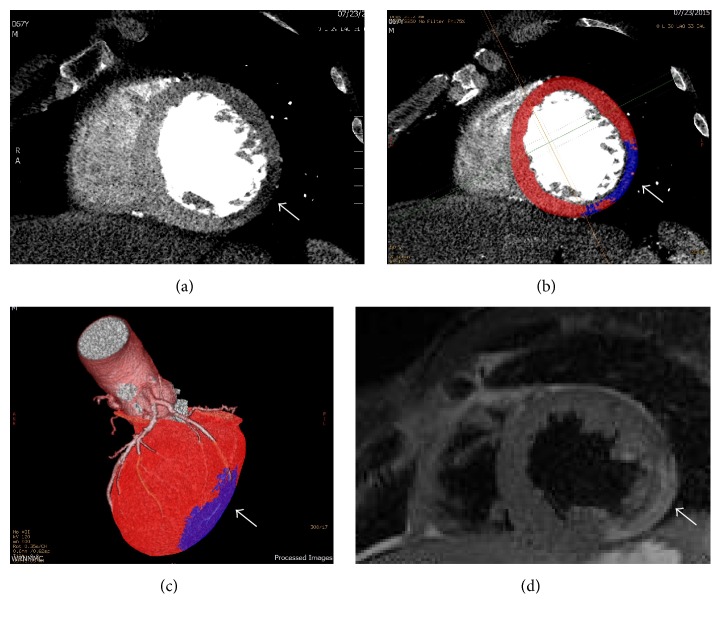
Short-axis first-pass CT perfusion images demonstrating an area of low attenuation of the mid inferolateral wall (a, b, c) corresponding to infarct on cardiac MRI delayed enhancement (d) images.

A 67-year-old male with a known bicuspid aortic valve was admitted with fevers and chest pain one month following a dental procedure. Upon initial presentation to an outside hospital, he was reportedly found to have an elevated troponin of 2.78 ng/mL and minor ST-segment elevations on ECG. Blood cultures drawn on admission grew alpha-hemolytic streptococci sensitive to penicillin and ceftriaxone. Both transthoracic and transesophageal echocardiograms demonstrated a subcentimeter aortic valve vegetation (Figure 1), moderate aortic regurgitation, and hypokinesis of the inferolateral left ventricular wall. The myocardial infarction was presumed secondary to septic coronary embolism, and invasive coronary angiography was foregone due to the embolization risk associated with the remaining vegetation. Instead, cardiac magnetic resonance imaging (MRI) was performed to better characterize the infarct for etiologic distinction. On cardiac MRI, the inferolateral left ventricular wall was found to have near-transmural late gadolinium enhancement (LGE) of the mid to apical portion, consistent with vasoocclusive myocardial infarction (Figure 2).

**Figure 3:**
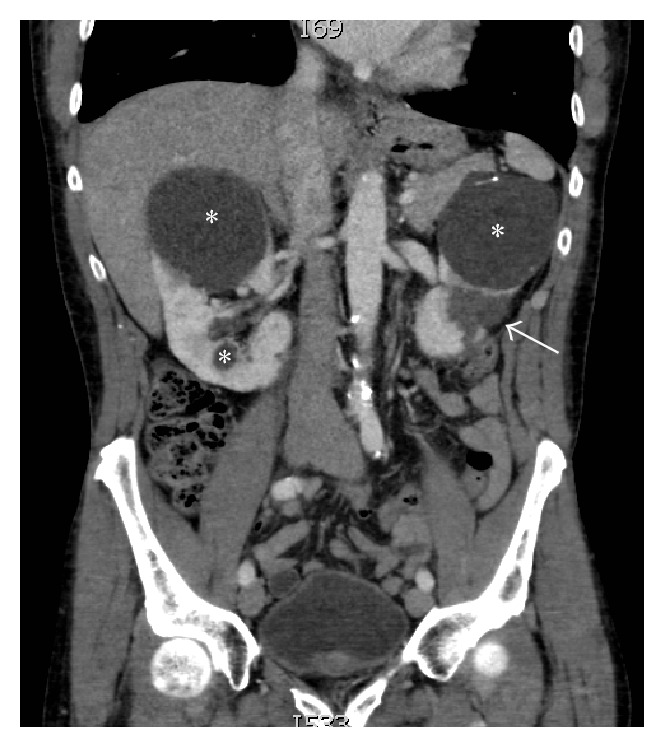
CT of the abdomen and pelvis with contrast, demonstrating a wedge-shaped area of hypoenhancement in the mid to lower pole of the left kidney (arrow), consistent with infarct from septic embolization. Incidentally noted were the large, bilateral renal cysts (*∗*), unchanged in appearance from prior imaging.

Due to vegetation size and the absence of heart failure symptoms, the patient was initially treated medically. A peripherally inserted central catheter was placed, and he was discharged on appropriate antibiotic therapy following negative repeat blood cultures. He subsequently returned five days later with persistent fever and new, severe left flank pain. Abdominal computed tomography demonstrated a wedge-shaped area of hypoattenuation consistent with a renal infarct (Figure 3). Blood cultures remained negative throughout this admission and repeat transthoracic and transesophageal echocardiography revealed no residual vegetation or perivalvular complications (Figure 1).

**Figure 4:**
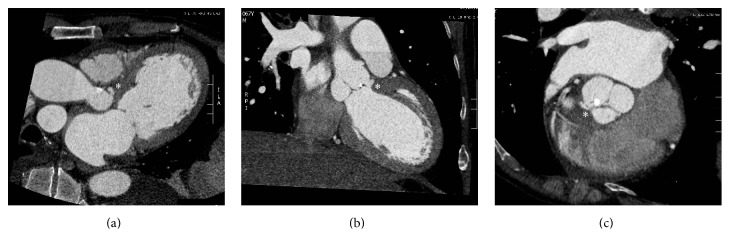
Multiplanar, reformatted oblique views of cardiac CTA (a, b, c) demonstrating the small pseudoaneurysm (*∗*) involving the aortic annulus and the mitral-aortic intervalvular fibrosa.

**Figure 5:**
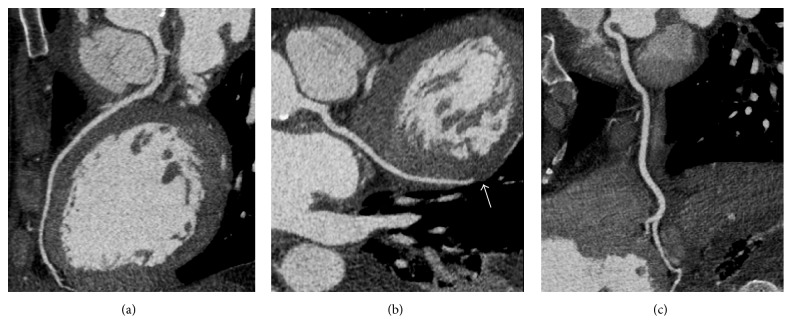
Curved multiplanar reformatted images of the left main and left anterior descending (a), left circumflex and terminal obtuse marginal branch (b), and right coronary arteries (c). There is an abrupt occlusion of the mid obtuse marginal branch (arrow) without significant coronary artery disease, suggesting that the coronary obstruction was likely embolic in etiology.

Cardiac computed tomography angiography (CTA) was then performed to better visualize potential perivalvular extension and provide concomitant assessment of coronary arterial disease. CTA was performed using a prospective ECG triggered acquisition using a 64-slice scanner (General Electric HD-750, Waukesha, Wisconsin, USA) during the administration of a triphasic contrast injection protocol (contrast, contrast-saline mix, and saline). CTA revealed an eight-millimeter mycotic aneurysm of the aortic annulus extending into the intervalvular fibrosa and no residual aortic valve vegetation (Figure 4). Additionally, there was occlusion of the mid portion of a terminal obtuse marginal artery, with a corresponding area of first-pass hypoperfusion in the inferolateral wall. With no evidence of underlying atherosclerotic disease (Figure 5), the aforementioned findings were determined to be most consistent with the diagnosis of embolic infarction. Based on CTA findings, the patient underwent successful aortic valve replacement and pseudoaneurysm repair, without the need for invasive coronary imaging or intervention.

## 3. Discussion

Mycotic aneurysms have widely varying clinical presentations depending on their location, the duration of infection, and patient comorbidities. The most common presenting symptoms are largely nonspecific, ranging from isolated leukocytosis or elevated erythrocyte sedimentation rate to bacteremia and fulminant sepsis [4]. This makes diagnosis a particular challenge, yet timely recognition and intervention are nevertheless critical in order to avoid life-threatening complications.

Due to exceedingly low incidence, little is known about the natural progression of mycotic aneurysms. Thrombosis, rapid enlargement, and even spontaneous regression are possible, though there are no known imaging findings that can accurately predict the clinical course [5]. The reported mortality of patients with aortic mycotic aneurysms, in particular, is estimated to be between fifteen and fifty percent. Rupture carries an exceptionally high mortality risk; thus, as in the case described, immediate surgical intervention is generally recommended [1].

CTA is the imaging modality of choice for the diagnosis of mycotic aneurysms [1]. By contrast, there is no definitive indication for the use of CTA in the diagnosis of infective endocarditis (IE). However, CTA has similar diagnostic accuracy to transesophageal echocardiography (TEE) for the diagnosis of IE, and particular studies have demonstrated its superiority to TEE for the detection of perivalvular extension [3, 6]. Additionally, current American College of Cardiology/American Heart Association guidelines for valvular heart disease state that CTA is reasonable to perform in cases of IE for which perivalvular complications are suspected, but inadequately visualized on echocardiography (Class IIA; level of evidence: B) [7]. Current appropriate use criteria also deem CTA appropriate for the assessment of coronary arterial disease prior to noncoronary cardiac surgery when catheterization is felt to be very high risk [8].

In this particular case, CTA identified a small but clinically relevant mycotic aneurysm that had not been visualized on either transthoracic or transesophageal echocardiography. The detailed assessment of both the coronary anatomy and the infarcted area allowed for confirmation of the embolic nature of the patient's MI and ruled out any atherosclerotic coronary disease requiring intervention during surgery.

Resting or first-pass perfusion deficits can often be detected in patients with acute or chronic infarction with any modern CT scanner (64-slice or more) equipped to do coronary CTA. Cardiac MRI performed 8–10 minutes following gadolinium contrast (delayed enhancement) is a well-validated technique to detect myocardial scar (fibrosis) from any etiology based on the known retention of contrast material in areas of fibrosis. Scar (areas of hyperenhancement on delayed imaging) in a coronary distribution, as seen in this case, is typical of a myocardial infarction from a coronary etiology. Similarly, an additional noncontrast cardiac CT scan performed 8–10 minutes following the initial cardiac CTA can also be done to assess for scar (hyperenhancement on CT), similar to cardiac MRI. In this case, a delayed CT scan was not performed due to the presence of the previously performed delayed enhancement MRI and to reduce patient radiation exposure.

Finally, this case highlights the advantage of cardiac CTA for coronary artery examination in lieu of high-risk catheterizations and nicely portrays its supplementary roles in both preoperative planning and the assessment of perfusion defects. It additionally demonstrates the utility of cardiac CTA for precise perivalvular assessment in infective endocarditis, particularly in cases of clinical suspicion for perivalvular extension and inadequate visualization on echocardiography.
